# Application of Hydrogels as Three-Dimensional Bioprinting Ink for Tissue Engineering

**DOI:** 10.3390/gels9020088

**Published:** 2023-01-19

**Authors:** Mengbo Xie, Jingjing Su, Shengxi Zhou, Jingan Li, Kun Zhang

**Affiliations:** 1 School of Life Science, Zhengzhou University, Zhengzhou 450001, China; 2 School of Materials Science and Engineering, Zhengzhou University, Zhengzhou 450001, China

**Keywords:** three-dimensional bioprinting, hydrogel ink, printability, fidelity, biocompatibility

## Abstract

The use of three-dimensional bioprinting technology combined with the principle of tissue engineering is important for the construction of tissue or organ regeneration microenvironments. As a three-dimensional bioprinting ink, hydrogels need to be highly printable and provide a stiff and cell-friendly microenvironment. At present, hydrogels are used as bioprinting inks in tissue engineering. However, there is still a lack of summary of the latest 3D printing technology and the properties of hydrogel materials. In this paper, the materials commonly used as hydrogel bioinks; the advanced technologies including inkjet bioprinting, extrusion bioprinting, laser-assisted bioprinting, stereolithography bioprinting, suspension bioprinting, and digital 3D bioprinting technologies; printing characterization including printability and fidelity; biological properties, and the application fields of bioprinting hydrogels in bone tissue engineering, skin tissue engineering, cardiovascular tissue engineering are reviewed, and the current problems and future directions are prospected.

## 1. Introduction

Total organ transplantation is currently one of the best ways to treat end-stage organ diseases. Although the number of organ donations has gradually increased in the past decades, the supply of solid organ transplants is still far from meeting the demand [1]. It is a pity that there were over 150 thousand candidates on the waiting list in Europe in 2018, but only 34,221 organ transplants were achieved that year [2]. During COVID-19‘s global pandemic, organ transplants increased the risk of patients contracting COVID-19 and progressing to serious illness [3]. After transplantation, many patients develop acute rejection within a year, and chronic rejection has a lifelong impact on patients. Creating autologous on-demand organs through biological manufacturing is a better way to solve organ deficiency and rejection [4]. Therefore, personalized artificial organs for disease treatment are urgently needed, but the great challenge lies in their manufacturing, which requires billions of cells to quickly organize into functional structural units [5].

Three-dimensional (3D) printing technology emerged in the 1980s and has been used in the direction of highly specialized demand, such as model making, and has become a multifunctional technology platform for computer-aided design (CAD) and rapid manufacturing [6]. This technology is a method to make 3D object rapid prototyping directly from CAD data, which is segmented according to a certain layer thickness and printed with specific materials through 3D printers with different functions [7]. The materials and products have great advantages in customizing shapes, customizing pore size/porosity, and adjusting mechanical properties [8]. It is more flexible, with less material and processing waste. Three-dimensional bioprinting enables unprecedented accuracy and precision by patterning cells and biological materials in 3D volumes in a highly repeatable manner supported by programmed robotic fabrication mechanisms [9]. It has been used to print tissue structures, organ modules, and organ-on-a-chip devices [10]. Hydrogels are important in 3D bioprinting due to their excellent water absorption, which can encapsulate nutrients and growth factors, mimic the natural tissue microenvironment, and achieve a high degree of biocompatibility [9]. They can be designed in any size, form, or shape [11]. With the progress of technology, many new 3D printing technologies and hydrogel bioinks have emerged, but there is a lack of systematic reviews on the functional materials of modified hydrogel inks and the latest technologies of 3D bioprinting. Therefore, this paper reviews the common polymers and their composite functions, the latest 3D printing techniques, characterization methods, biological properties, and their applications in the field of tissue engineering.

## 2. Hydrogel Inks and Technologies of 3D Bioprinting

**Figure 1:**
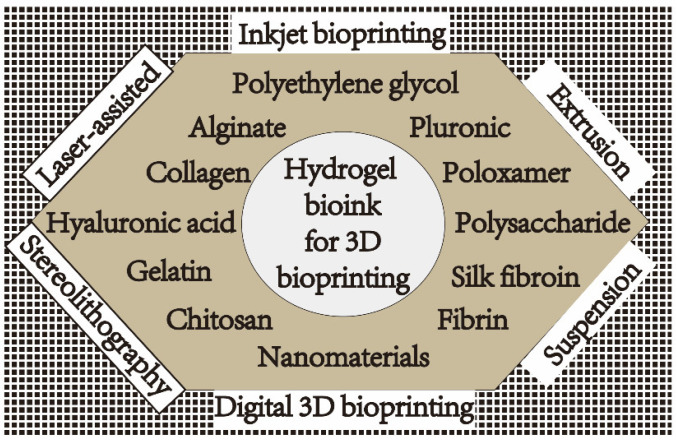
Materials and technologies for 3D-bioprinted hydrogels.

In the process of 3D bioprinting, hydrogel materials, bioprinting technology, and cells are closely related. Bioprinting technology has increased exponentially since it came out because it can print complex structures simply and quickly [12]. The materials of hydrogels using different bioprinting technologies also tend to be diversified as shown in Figure 1.

### 2.1. Materials as Hydrogel Inks

The sources of hydrogel materials are mainly divided into polymers and modified polymer complexes. The former are natural or artificial polymers which can form hydrogels, while the latter are polymer composite inks constructed by adding functional molecules on the basis of them. Natural hydrogels currently used for bioprinting mainly include collagen, gelatin, alginate, fibrin, silk, chitosan, hyaluronic acid, etc. Collagen, the most abundant extracellular matrix protein and a commonly used bioprinting material, has been widely used in tissue engineering due to its unique biocompatibility and low immunogenicity [13]. Gelatin, the second most commonly used material, is a natural protein with amphiphilic behavior due to basic and acidic amino acid functional groups, originating from collagen hydrolysis [14]. It has great potential in the field of bioprinting due to its water solubility, biocompatibility, biodegradability, handleability, low cost, and immunogenicity [15,16]. Alginate hydrogels, as one of the most popular natural materials, exhibit gelling abilities, low toxicity, high availability, and a low cost [17]. For example, 3D bioprinting technology based on gelatin/alginate hydrogel is used to prepare a multilayer composite scaffold with stratum, corneum, and dermis simulating the hair follicle microenvironment in vivo [18]. Fibrin hydrogels, with their biocompatibility, biodegradability, and tunable mechanical and nanofibrous structural properties, have been widely used in tissue engineering and more recently in 3D bioprinting [19]. Silk fibroin is a natural protein with excellent mechanical properties, biodegradability, biocompatibility, and bio-absorbability in bioprinting hydrogel materials, which has attracted great attention in many fields [20]. A methacrylic light-curable silk fibroin bioink for digital light processing 3D biological printing has been developed to produce a high mechanical stability, biocompatibility, and accurate printing for tissue engineering applications [21]. Chitosan, a polycationic biocompatible natural polymer, whose solution is stable and viscous under physiological conditions, forms a 3D printing scaffold to support cell proliferation and differentiation [22]. For example, a chitosan self-healing hydrogel for injectable and printable inks was prepared using phenol functionalized chitosan and dibenzaldehyde capped by distal chelating polyethylene glycol. The phenol functionalization of chitosan can introduce unique interactions that enable hydrogels to have fast gelation rates, self-repair abilities, and distant critical gel behavior, as well as secondary visible light crosslinking abilities [23]. Hyaluronic acid, a natural linear endogenous polysaccharide, has important biological characteristics such as biocompatibility, biodegradability, and non-immunogenicity, so it is widely used in hydrogel scaffold materials [24]. For example, a new type of composite hydrogel ink was prepared by rapidly forming a dynamic covalent bond between phenylboric acid-grafted hyaluronic acid and polyvinyl alcohol and further stabilizing gelatin by secondary crosslinking between the acrylate part of phenylboric acid-grafted hyaluronic acid and the mercaptan-free group [25]. After the addition of carrageenan, the alginate/carrageenan composite hydrogel has an enhanced rheological behavior and can be used in extrusion-based bioprinting [26]. In short, these polymers come from natural materials that usually have biocompatibility.

Ideal materials usually have excellent mechanical properties, biodegradability, a controllable degradation rate, and nontoxic degradation products [27]. Artificial polymers play a key role in supporting cell and biomolecule activity in 3D printing. Their networks comprise repeatable inert units, and their properties in terms of mechanical properties and immunogenicity are usually superior to those of natural polymers [28]. For example, polyethylene glycol (PEG), an artificial polyether with a linear and branched structure, has non-immunogenicity, hydrophilicity, and biocompatibility. It is a well-known artificial biomaterial, commonly used as a photo-crosslinked bioink for bioprinting. A novel molecularly engineered microcapillary-based PEG-based bioink for bioprinting was prepared that transiently binds low molecular weight gelatin fragments [29]. Poloxamer is a triblock copolymer synthesized from polyoxyethylene-polypropylene oxide-polyethylene oxide; bioinks based on poloxamer 407 and PEG blends provide hydrogels with different yield stresses [30]. Thermal responsive Pluronic/alginate semisynthetic hydrogel was used for bioprinting 3D liver structures, which had the characteristics of high shape fidelity, moderate deposition conditions, and controlling the gelation mechanism. In addition, the dissolution of sacrificial Pluronic templates significantly improved the diffusivity of printing hydrogels [31]. Polysaccharide hydrogel composites of nanocellulose, agarose, and sodium alginate were used to encapsulate cells as biological inks; polyvinyl alcohol was used as a sacrificial material to construct noncollapsing structures such as lips and noses, and nutrient networks gradually formed vascular structures for personalized, complex, and vascularized tissue engineering [32].

Bioprinting is used to facilitate the fabrication of engineered tissue for patient-specific defect repair and to develop promising technologies for in vitro tissue/organ models for ex vivo testing. However, due to the inherent swelling/shrinkage and bioinert properties of most polymer hydrogels, polymer-based ink materials typically result in low scaffold fidelity, insufficient mechanical strength, and loss of osteogenesis induction. Functional molecules are often used to improve the performance of biological inks. For example, human mesenchymal stem cells loaded with graphene oxide/alginate/gelatin composite bioinks were developed to form 3D bone-like scaffolds using 3D bioprinting technology [33]. The main challenges of tissue manufacturing using hydrogels are their lack of regeneration, uncontrolled expansion, degradation, and their inability to maintain 3D structures on their own. Nanomaterials, including nanoparticles, nanofibers, and nanospheres, are commonly added to various bioinks, giving tissue-engineered scaffolds new properties such as magnetism, electrical conductivity, and mechanical strength [34,35]. For example, SiO_2_ nanoparticles can interact with alginate–gelatin polymer networks and are embedded into alginate/gelatin hydrogels as patient-specific virtual defects to make nano-particle hydrogel composite inks for bone grafts [36].

The materials of 3D printing hydrogel inks mainly come from natural polymer materials with biocompatibility, and artificial polymers or nanomaterials are usually used to improve their mechanical properties, printing fidelity, and other properties to meet application requirements.

### 2.2. Technologies of 3D Bioprinting

The methods of synthesizing hydrogels using bioprinting technology also tend to be diversified; 3D bioprinting technologies mainly include inkjet bioprinting, extrusion bioprinting, laser-assisted bioprinting, stereolithography bioprinting, suspension bioprinting, and digital 3D bioprinting technologies, etc. 

#### 2.2.1. Inkjet Bioprinting

Inkjet technology, capable of producing droplets in the picolitre volume range, jetting thousands of times in seconds, and printing noncontact, has been widely used since its inception and is currently used in medicine for drug development, scaffold construction, and cell deposition, etc., and has also made important breakthroughs in the field of bioprinting [37]. Inkjet printing is capable of being compatible with many biological materials and printing through the same structure with different cell densities by varying the droplet density or size [38]. For example, the advantage of on-demand droplet inkjet printing lies in the use of interfacial fluid forces to guide the self-assembly of cells, peptide amphiphiles, and a variety of extracellular matrix proteins and biomolecules into arranged or disordered nanofibers, hydrogel structures with different geometric shapes and sizes, surface morphologies, and high-order structures constrained by molecular diffusion. The combined NIH-3T3 and adipose-derived stem cells have a high cell viability in the complex structure of bioprinting [39]. It can produce ink droplets of controllable size and transport them to a specific location, with the characteristics of low cost, high cell vitality, and high speed. However, there are few materials and crosslinking agents suitable for hydrogel bioinks at present due to the requirements of inkjet bioprinting on biocompatibility, mechanical properties, fluidity, and viscosity of the bioinks. The design of nozzles, including the size and number, limits the concentration and number of cells [37,40].

#### 2.2.2. Extrusion Bioprinting

Extrusion bioprinting is a successful 3D printing method in tissue engineering and biological manufacturing [41]. The direct writing-based extrusion bioprinting machine works by loading the bioink into a syringe barrel and then extruding it through the tip of a micronozzle. The method is simple, economical, and expansible and is widely used at present [42]. A multi-material extrusion biological printing platform was developed. The platform enables the continuous deposition of multiple coded bioinks, rapid and smooth switching between different reservoirs for the rapid fabrication of complex structures, and digitally controlled extrusion of bioinks from a single print head consisting of bundled capillaries in coordination with the programmed motion of a motorized table [43]. It is beneficial to improve the printability and fidelity to create 3D structures with preferred shapes and forms by improving the viscosity of the bioink. However, the viscosity not only increases the shear stress and weakens the cell compatibility, but it also increases the flow resistance, which may cause nozzle blockage [44]. Additionally, the resolution is limited, and it is especially difficult to achieve submillimeter resolutions [45]. Due to the changes in nozzle geometry, printing time, dispensing pressure, and biological ink concentration, the cell vitality is low and the material is missing [46].

#### 2.2.3. Laser-Assisted Bioprinting

Laser-assisted bioprinting is also named laser-induced forward transfer bioprinting. In contrast to inkjet bioprinting and extrusion-based bioprinting, laser-based bioprinting is nozzleless, which makes it an efficient tool that can adapt to the viscosity of the bioinks [47]. Because of its automation, high cell survival, high resolution, and good accuracy, laser biological printing technology can print biological ink in situ so as to promote tissue regeneration and create 3D-defined precancer and cancer models. For example, different combinations of acinar and/or ductal exocrine pancreatic cells were replicated to form spheroids with laser-assisted bioprinting, which constituted a 3D model suitable for studying the initial stages of cancer development. However, the workstation that requires a laser source to participate in construction is complex [48].

#### 2.2.4. Stereolithography Bioprinting

Stereolithography is another well-established method capable of producing high-resolution structures with a high dimensional accuracy and smooth surface quality of printed parts, enabling the removal of uncured resin from the final product [41,49]. A rapid hydrogel stereolithography method has been proposed, which can accurately control the photopolymerization conditions, establish the low suction drive and high-speed flow of the hydrogel prepolymer, and support the continuous replenishment of the prepolymer solution under the curing part and the uninterrupted growth of the part. Using the stereolithography method to allow rapid printing of centimeter-sized solid hydrogel models in a few minutes can significantly reduce part deformation and cell damage caused by long-term exposure to environmental stress in traditional 3D printing methods. The embedded vascular network manufactured by multiscale printing allows for the medium perfusion needed to maintain high cell viability and metabolic function in the deep core of the large model, which provides the possibility for the construction of large-scale hydrogel engineering tissue models [50]. However, its ability to capture the spatial-heterogeneity that permeates mammalian tissues and determines structural–functional relationships is limited [51].

#### 2.2.5. Suspension Bioprinting

Infiltration-induced suspension bioprinting is a novel printing technology based on the suspension system of hydrogel materials, such as hyaluronic acid. Because of its self-healing rheological and shear thinning properties, HA is simple to prepare, reusable, and it is easy to adjust its osmotic pressure [52,53]. The change in osmotic pressure can guide the expansion or contraction of bioinks based on the 3D-printed gelatin methylacryl (GelMA), which can adjust the physical characteristics of the 3D-printed scaffold including its micromorphology, fiber diameter, water absorption, and mechanical strength. A scaffold containing human umbilical vein endothelial cells with resolution and cell viability was prepared using infiltration-induced suspension bioprinting technology, which is expected to meet the requirements of scaffolds [54]. Suspension baths can take into account the printability and biological activity of biological ink, broaden the range of materials suitable for 3D biological printing, and are not limited by the printing position, thus forming a complex structure. However, the printing temperature is determined by the temperature of the suspension medium [55,56].

#### 2.2.6. Digital 3D Bioprinting

**Figure 2:**
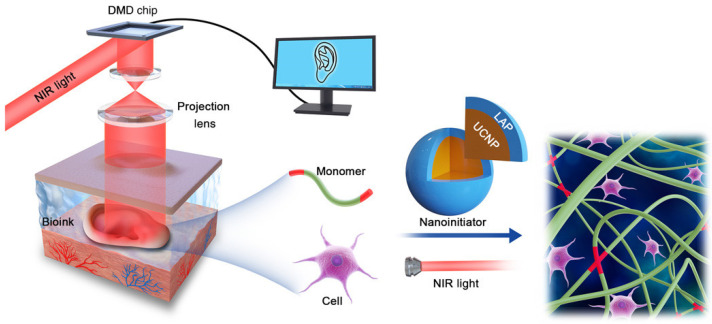
Schematic diagram of DNP-based noninvasive 3D bioprinting [60].

Bioprinting is a powerful tissue engineering application technology that enables the design and simulation of different tissues and organs through digital control, and its high throughput and precise control of scaffolds and cells are valuable among the many advantages reported by bioprinting. Digital-based 3D bioprinting technology has developed rapidly recently, such as digital light projection (DLP), digital assembly of spherical particles (DASP), and digital near-infrared photopolymerization (DNP). The emergence of 3D printing technology based on DLP has greatly promoted the biological manufacture of photopolymerized hydrogel biomaterials. In particular, this technology can achieve higher spatial resolutions in the micron scale (3–5 μm) and faster printing times in the order of seconds [57]. DLP-based 3D bioprinting technology provides a high resolution and spatiotemporal control, and it is used to prepare microscale acellular and cellular GelNB-GelSH constructs [58]. Based on the advances in embedded printing, a 3D bioprinting technology has been developed which allows for DASP. Different from existing 3D printing technology based on droplets, DASP can generate, deposit, and assemble viscoelastic bioink droplets on demand in a cell-compatible environment [59]. At present, the in vivo application strategy of 3D printing macro products is limited to surgical implantations or in situ 3D printing of exposed trauma, both of which need to expose the application site. Therefore, a 3D printing technology based on DNP was developed, which can perform noninvasive 3D biological printing of tissue structures in vivo. (Figure 2) In this technology, the customized CAD model data is sent to the DMD chip. The digital micromirror equipment is modulated into a customized pattern by near infrared and is dynamically projected to induce the polymerization of the monomer solution in space. Through the use of patterned near-infrared external irradiation, the subcutaneous injection of biological ink containing UCNP@LAP nano-initiator can convert near-infrared light into 365 nm light, thus initiating the optical mode-controlled polymerization of monomers and noninvasive in situ printing into customized tissue structures [60]. Fine structures with multicell-laden hydrogels can be manufactured. However, more accurate fine tissues constructed in situ require more complex workstations, high-precision instruments, and composite functional hydrogel biological ink materials [56].

Their advantages and disadvantages are summarized in Table 1. With the advances in technology, 3D biological printing technologies emerge one after another, and some new methods are being invented. The ultimate goal of these methods is to ensure cell activity, printing fidelity, and the long life of the scaffolds to meet the clinical needs of tissues or organs.

## 3. Printing Characteristics and Biological Properties of 3D Bioprinting Hydrogels

Three-dimensional bioprinting is an additive manufacturing technique for building complex tissues and organs [61]. Hydrogels have porous structures that can encapsulate and support different cells and active substances. Whether the crosslinking mechanism of hydrogels is physical and/or chemical, it is generally required to have stable printing characteristics (such as density, viscosity, fluidity, and deformability) and to be compatible with organisms (nontoxic, degradable, and adhesive and porous) [62,63,64]. Therefore, it is necessary to systematically study the overall characteristics of 3D-printed hydrogels to determine their applicability.

Printing fidelity is the degree to which printing is carried out according to a CAD plan. Printing fidelity typically refers to the characteristics of the printed specimens, and it is usually characterized by the diameter, uniformity, angle, and area of the printed strands. The three-dimensional bioprinting of hydrogels with sufficient structural and shape fidelity has been a challenge due to the inherent flow behavior and weak mechanical properties of hydrogels, especially in the fabrication of large clinical-scale tissue constructs [65]. It is a key parameter used to describe the bioprinting properties of bioinks. Cationic modified silica nanoparticles are added to the anionic polymer mixture composed of gelatin and alginate. Because of the electrostatic interactions between nanoparticles and polymers, nanoparticles can significantly inhibit the shrinkage and swelling of printing structures during crosslinking, which leads to high printing fidelity [66]. The viscosity, shear thinning, and thixotropic behavior of 3D bioprinting hydrogels can be measured by rheology. Viscosity is a basic characteristic of biological ink, which affects printing quality and processing as well as cell viability. Frequency scanning has been used to measure the viscosity of bioinks at different shear rates [67,68]. The effect of electric charge on 3D biological printing can be reflected in the effect on the viscosity and the shear rate of the bioink. At the same shear rate, the viscosity of bioinks is the highest for anionic polymers, the lowest for cationic polymers, and medium for nonionic polymers [44]. Hydrogel precursors usually exhibit non-Newtonian behaviors, so the shear stress–shear rate curve obtained by the shear rate scan is nonlinear and concave, which is characterized by a low viscosity. Low-viscosity materials may have adverse effects on printing pressure control [69]. Based on rheological properties, a high viscosity at low shear rates enables the developed bioinks to maintain a stable structure after leaving the printing nozzle. However, excessive viscosity usually limits cell survival and function. Therefore, a delicate balance should be struck between the printing fidelity and cellular compatibility of bioinks to achieve optimal printing efficiency [70,71]. A generic rheological model can be used to make viscosity data compatible with finite element methods and other simulation programs. Different rheological model results provided by bioinks can be used to further optimize the printing process of 3D bioprinting hydrogels. Ideally, the hydrogel 3D printing process involves three stages: (1) structural evolution of the bioink from a liquid state to a gel state during extrusion; (2) to form a printing layer and adhesion between layers on the substrate to print multilayer structures; and (3) the self-supporting stage of structural recovery [72]. Axel et al. used rheological analysis to evaluate the printing behavior on a wide range of key process parameters of extrusion-based bioprinting to enable the identification of hydrogels in liquid, gelatinous, and intermediate states [73]. The elastic modulus is another important property that determines the deformation of 3D structures under bending. It also affects the printing ability because less-elastic compounds are too hard and break in the nozzle, while the more-elastic compounds are too soft [74]. A series of hydrogels with tunable modulus mechanical properties were developed as bioinks, such as polyurethane–gelatin, dopamine-functionalized GelMA, and acrylate β-cyclodextrin, and are more helpful to obtain shape fidelity [75,76]. In addition, the 3D bioprinting hydrogel structure, pore size, and porosity can also be observed through scanning electron microscope images [77]. In practical applications, hydrogels need to have a high porosity to support oxygen and nutrient delivery. In addition, the communication between pores is also conducive to promoting cell migration and angiogenesis [78]. Therefore, structural factors such as pore size have been used to characterize the printability of hydrogels. In order to evaluate the structure more accurately, the promising imaging techniques of microcomputed tomography, X-ray propagation-based imaging in combination with CT imaging, have been used to reconstruct the entire 3D structure of bioprints and analyze porosity [79].

Hydrogels made by 3D bioprinting generally have biocompatibility, biodegradability, and a relatively low cost. To enhance their biomimicry, 3D printing hydrogels are often combined with cells, growth factors, cytokines, and other molecules to suit specific biological applications, as shown in Table 2.

3D-bioprinted hydrogels provide cells with suitable microenvironments, such as CCD-986Sk cells which showed activity in sodium alginate-xanthan gum@ carboxylated-cellulose nanocrystal (SA-XG@cCNCs) hydrogel which provided living space for the cells and could transport nutrients into the cells [80]. Similarly, methylcellulose/alginate hydrogel can maintain stable activity for 7 days, indicating that the hydrogel can support cell survival [81]. The cells in gelatin–alginate hydrogel also remained active and exhibited an increased cell proliferation compared to the two-dimensional culture group [18]. Cells in alginate, fibrin, and GelMA hydrogels differentiated and increased the microvascular stability of human umbilical vein endothelial cells (HUVECs) and human bone marrow mesenchymal stem cells with different proportions [83]. These biological and physical characteristics indicate that bioinks can print dynamic and personalized biological structures and can be matured in vitro in xenogeneic environments [84]. The results of interleukin-4 coated with GelMA-dex hydrogel and human mesenchymal stem cells indicate that this bioink has antibacterial and potentially anti-inflammatory properties, which provides a new method for suppressing bacterial infections and immune regulation [85]. Since cartilage regeneration typically takes a long time, 3D bioprinting hydrogels with a long-term stability and mechanical integrity would be beneficial for this purpose [21]. By optimizing the fibrinogen concentration, modification, crosslinking method, etc., stable fibrin hydrogels can be prepared, showing cytocompatibility, promoting cell attachment, diffusion, and proliferation on the hydrogel so as to facilitate the embedding of human chondrocytes providing enough time to form new cartilage [88]. Hydrogels can protect cells from shear stress-induced cell membrane damage during 3D printing and can improve cell viability in printed structures [89], which provides a suitable 3D microenvironment for cell adhesion, proliferation, and migration [80,90]. A hydrogel containing aspartic acid was prepared by Motealleh et al. This hydrogel has a strong mechanical structure and allows the tissue structure to have large pores, which can effectively improve cell adhesion, migration, proliferation, growth, tissue, oxygen, and nutrition transport [91]. Therefore, 3D-bioprinted scaffolds can ensure the survival of the vast majority of cells and allow them to proliferate, differentiate, undergo phenotypic changes, and function, and the development of printable, fidelity, and biocompatible 3D printing scaffolds remains the focus of characterization, but it is still far from clinical needs.

## 4. Application of 3D Bioprinting Hydrogels

3D bioprinting has become a promising technology, which combines cells with hydrogel inks to generate tissue-like structures through layer-by-layer manufacturing methods, simulating the microenvironment of tissues and cells for tissue engineering repair and reconstruction [92]. This part mainly summarizes the application of bone, skin, and cardiovascular tissue engineering.

### 4.1. Bone Tissue Engineering

Bone is a complex structure that consists of hierarchical tissue and mineralized collagen fibers, a vascular system, etc. [93]. Bones have a strong regenerative capacity and are able to repair small cracks and fractures on their own. However, when there is a bone defect larger than two centimeters, the bone cannot repair itself [94]. Bone defects need to be reconstructed using custom-made grafts to restore structure and function. However, traditional hydrogels lack precise control over the internal structure as well as the distribution of growth factors, etc., and in order to overcome these shortcomings, innovative methods of 3D bioprinting have been introduced [14].

**Figure 3:**
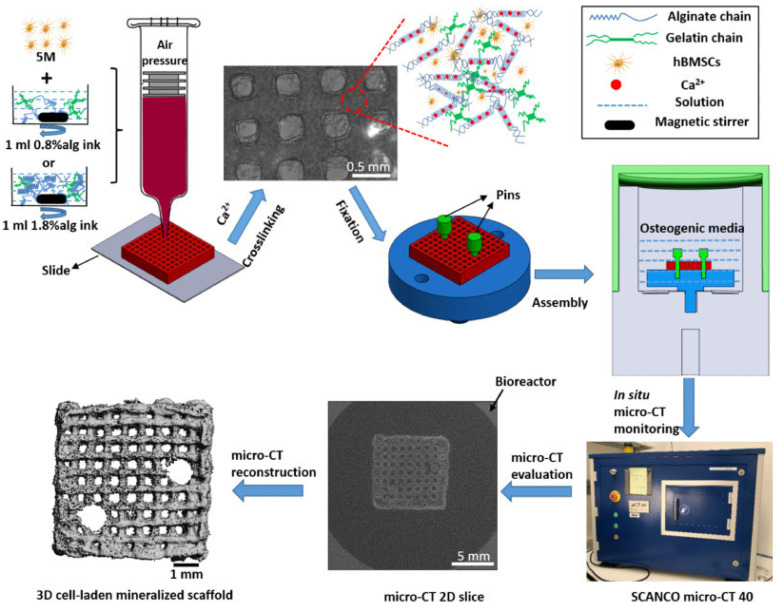
Alginate bioink for 3D bioprinting for bone tissue engineering scaffolds [97].

Using 3D bioprinting to process stem cell-bearing biomaterials opens up potential for bone tissue engineering to create living 3D structures [95]. Crosslinked hydrogel inks based on calcium ions are widely used, such as sodium alginate, GelMA, etc. For example, Marcia et al. developed a formulation of a GelMA/MSNCaPDex hydrogel bioink with biocompatibility and potential for 3D bioprinting stem cell-bearing structures [96]. Based on extruded 3D bioprinting technology, alginate, gelatin, and human mesenchymal stem cells were combined to form a simple and low-cost biological ink for the manufacturing of 3D bone-like tissues containing osteoblast tissue [97] (Figure 3). Hernandez-Gonzalez, A.C., et al. reported a nuclear/shell structure scaffold consisting of calcium-deficient hydroxyapatite and a pre-osteoblast MC3T3-E1-loaded alginate hydrogel, and the 3D-printed design includes a metal core nozzle covered by an external nozzle that extrudes the housing under pneumatic and mechanical pressure [98]. Cidonio, G. et al. used Laponite^®^–alginate–methylcellulose to inoculate human bone marrow stromal cells as bioink for generating bone mineral tissue in vitro and in vivo [99]. Three-dimensional bioprinting can combine biological components (especially collagen) with supporting hydrogels to increase stiffness; bone marrow mesenchymal stem cells show osteogenesis differentiation abilities in agarose collagen hydrogels with low hardness and do not limit the stretching and branching of cells [100]. In addition, Yuan, W. et al. combined silica nanoparticles with surface-modified methacryloyl groups in gelatin host–guest hydrogels in light crosslinking; this hydrogel can accelerate the diffusion of stem cells wrapped in it and enhance the mechanical sensing ability of the stem cells, thereby enhancing the bone-forming ability of the stem cells [101]. In addition, based on silica, Monavari, M. et al. added mesoporous silica–calcium oxide nanoparticles into alginate dialdehyde–gelatin hydrogels that not only enhance the mechanical strength of the hydrogel structure, but also promote cell adhesion and proliferation, and at the same time, the hydrogel can release icariin to promote osteogenesis [102]. However, in the process of drug release, the pores inside traditional hydrogels have a great influence on the drug release carried by them. Therefore, Gupta, D. et al. combined 3D printing and freeze-drying technology to prepare a gelatin–gellan gum composite scaffold, which has a complex shape with multiscale porosity. The performance of loading antibiotics, cells, etc., is enhanced, which can better promote bone formation [103]. In conclusion, many achievements have been made in the application of hydrogels as bioinks for bone tissue engineering. However, there are not enough types of hydrogels to match the mechanical properties of natural bone, and it is still difficult to easily achieve high mineralization of bone tissue and proliferation of cell diversity during application. Many hydrogel-loaded stem cells are used as bioinks to promote bone regeneration by 3D bioprinting, but new materials and methods are needed to meet the requirement of differentiation.

### 4.2. Skin Tissue Engineering

Skin is the organ that protects the body from the environment, microorganisms, parasites, heat, ultraviolet rays, and water loss [104]. Meanwhile, skin-related diseases are also listed as the fourth most nonfatal disease in the world, affecting about one-third of the world’s population [105]. Normal wound healing includes a series of events, such as hemostasis, inflammation, proliferation, and extracellular matrix remodeling [106]. Traditional wound dressings can protect wounds from contamination, but these dressings need to be changed frequently [107]. In addition, they limit the movement of human joints, thus causing great inconvenience to patients [108].

**Figure 4:**
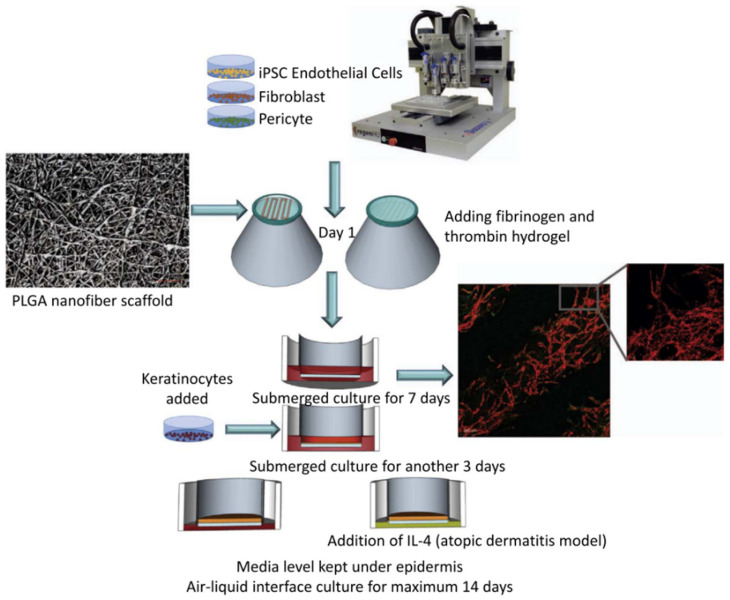
Bioprinting process of vascularized full-thickness skin [117].

The application of 3D bioprinting methods in wound healing and skin regeneration began in 2009 [109]. Hydrogels have become the best choice of bioinks for 3D bioprinting due to their biocompatibility and degradability [110]. There are many studies on the use of 3D-printed hydrogels as biological scaffolds for skin injury treatment. Highly ductile and highly elastic biocompatible scaffolds prepared from catechol-modified hyaluronic acid and alginate as materials are reported [111]. Using aminooxy-terminated Pluronic F127 and oxidized dextran as materials toughened by low temperatures (about 16 °C) and high temperatures (37 °C), the 3D-printed hydrogel has a high toughness and biocompatibility and can support the adhesion and proliferation of skin cells [112]. In the study by Zidaric, T. et al., nano-fibrillated cellulose was combined with alginate, carboxymethyl cellulose, and human-derived skin fibroblasts to produce a bioink that made it possible to print complex skin structures similar to the original dermis of the skin [113]. The alginate/gelatin composite hydrogel prepared by Liu, P., et al. also underwent a two-step gelation mechanism and was loaded with human amniotic epithelial cells with superior epithelial cell phenotype and Wharton gum-derived mesenchymal stem cells with excellent angiogenesis potential and fibroblast phenotypes [114]. Three-dimensional-printed hydrogels can not only be used as biological scaffolds, but also can be loaded with antibacterial drugs for the targeted treatment of skin lesions. In a recent study, a carboxymethylcellulose–human keratin hydrogel coated with clindamycin, which has cytocompatibility and a cell survival rate of more than 90%, was prepared by physically and chemically characterizing it to indicate its applicability to the application of skin dressings [115]. Rastin, H. et al. designed a hydrogel based on methylcellulose/alginate, and gallium was used in the formulation of this novel bioink, which can crosslink with Alg to stabilize the hydrogel, while the crosslinked hydrogel exhibited a strong antibacterial activity with a sterilization rate of 99.99% [81]. Si, H. et al. introduced a novel hyaluronic acid-based hydrogel for bioprinting, which is prepared by mixing the indiscriminate methacrylic anhydride and 3,30-dithiobis (propionylhydrazide) and incorporating Naficillin into the hydrogel, which proved to be a prospect in wound repair by comparing the drug release curve and cytocompatibility of the hydrogel [116]. In addition, endothelial cells were derived from induced pluripotent stem cells, fibroblasts, pericytes, and human keratinocytes and were used to produce skin-equivalent tissues with various physiological complexities, including human epidermis and full-thickness skin equivalents for drug screening. (Figure 4) The obtained skin model has layered structure markers of dermis and epidermis and has the physiological function of the skin barrier. Therefore, the physiologically close atopic dermatitis disease model in this study can be used to quickly understand the pathological mechanism and test the efficacy and toxicity of drugs [117]. It can be seen that 3D-printed hydrogels, as the carriers of drugs or cells, have a broad prospect in the application of skin injury treatment. Hyaluronic acid and sodium alginate are widely used as hydrogel inks in the construction of skin tissue engineering, but it is difficult to fully realize the construction of artificial skin because of the complex structure and appendages of skin. It is necessary to explore new biological hydrogel inks and printing technologies to construct orderly tissue-engineered skin from multiple dimensions of time and space.

### 4.3. Cardiovascular Tissue Engineering

The cardiovascular system consists mainly of the heart, blood vessels, and lymphatic vessels [118]. Cardiovascular diseases currently account for one of the highest numbers of deaths in the world each year due to the high prevalence of coronary artery disease, the sharp rise in population ageing, and the continued rise in obesity [119], with 23.3 million people expected to die annually by 2030 [120]. Current treatments for severe cardiovascular diseases are vascular grafts for bypass surgery to replace damaged blood vessels, but autografts are often unusable and have the potential to cause secondary harm to patients [121]. Thus, many technologies have been developed to mimic the manufacturing of human vascular network systems, and 3D bioprinting technology has become an important tool for the manufacturing of vascular biochemical structures due to its advantages, such as the control of vascular growth, the scalability of the manufacturing process, and the repeatability [122].

**Figure 5:**
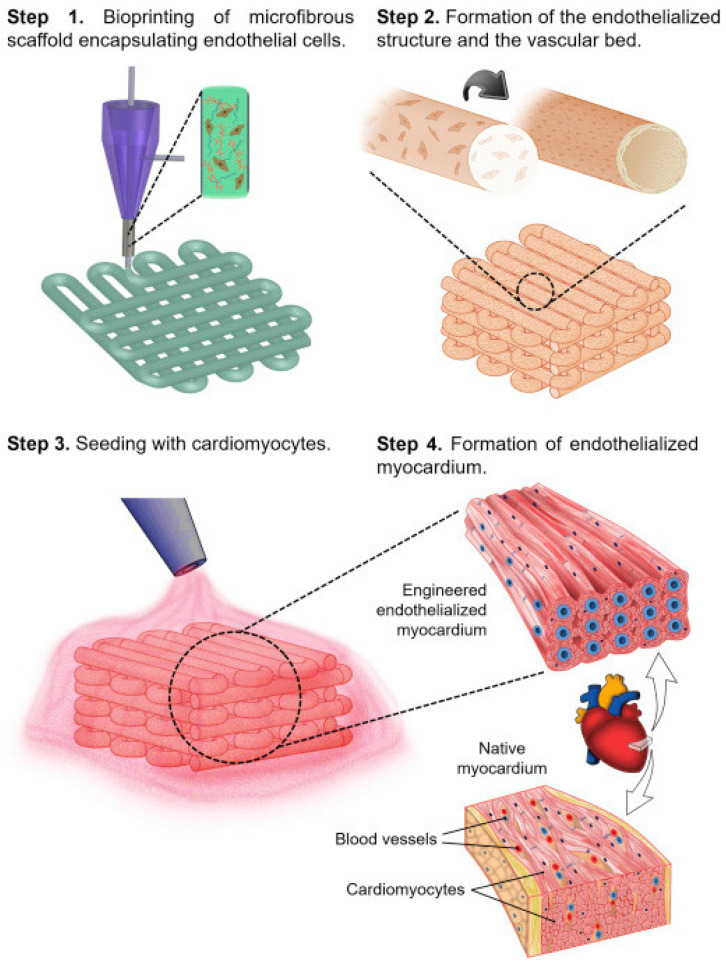
A schematic diagram of the process of constructing endothelialized myocardium based on 3D bioprinting [129].

The composition of bioinks affects various characteristics of the printed structure, and hydrogels such as alginate and gelatin show great potential when used as biomaterials for 3D bioprinting applications [123]. Liu, Y. et al. fabricated a polycythane–alginate printable dual-network hydrogel loaded with HUVECs, a highly mechanical hydrogel that can be bioprinted as artificial small-diameter blood vessels, and the total height of the model was 12 mm [124]. In the study by Zhang et al., alginate hydrogels containing HUVECs were printed by coaxial 3D printing, forming hollow filaments by crosslinking [125]. The three-dimensional-printed core microstructure of the alginate core with a biocompatibility of different sizes and traits was further embedded in the bio-support material and HUVECs were sowed after optical crosslinking and immersion in EDTA solution and were colonized to form a perivascular network [126]. In addition, 3D-printed hydrogels can also be used as cardiac patches to treat cardiovascular disease using aerosol jet printing two-dimensional titanium carbide MXene-hydrogel composites to design human heart patches [127]. Methacrylate gelatin/polyethylene glycol diacrylate/alginate, through light crosslinking, can be used for adjuvant treatment of heart valve disease [128]. A 3D-bioprinted hydrogel scaffold was prepared by using the two-step crosslinking method, in which alginate components were physically crosslinked by CaCl_2_ and then GelMA components were chemically crosslinked by ultraviolet light. (Figure 5) It can accurately control the large-scale anisotropic structure of microfibers and promote induced pluripotent stem cells to form an endothelialized human myocardial model. When further combined with a designed microfluidic perfusion bioreactor, the myocardial platform on the endothelial chip is used to screen the cardiovascular toxicity of drug compounds [129]. It can be seen that the use of 3D-printed hydrogels for the treatment of cardiovascular diseases has a broad prospect. However, 3D-bioprinted patient-specific heart patches to treat heart failure are still in the preclinical stage. Given the complex structure of heart muscles with artificial blood vessels, 3D-printed hydrogels still have a lot of room to search for heart-specific bioinks to meet cell heterogeneity and cardiac function requirements. From the perspective of application, processability and printability, high fidelity, and biocompatibility are the key points that need to be paid attention to at present. Due to the inherent instability of hydrogels, the fidelity of printing is affected. It is important to find a balance between the two and to create a 3D hydrogel structure with high fidelity and cytocompatibility. Powerful platforms with modern 3D bioprinting technologies can further expand the application of bioprinting soft tissue structuresw.

## 5. Conclusions and Respective

This paper mainly reviews the development of hydrogel inks and technologies, printing characterization, and biological properties of 3D-bioprinted hydrogels. As an integration of various innovative technologies, the 3D bioprinting technology has broad prospects in tissue engineering applications. There is hope to alleviate the high demand for organ transplants and the low supply of available organ donors [130]. With the accelerated aging of the population and the increase in human life expectancy, the demand for biosynthetic materials will further increase, and 3D bioprinting will increasingly play an important role in tissue engineering and regenerative medicine [131]. However, the current structural complexity and structural accuracy of 3D bioprinting are far from meeting the needs [132]. As one of the irreplaceable elements of 3D bioprinting, bioink has recently attracted investment. In 2016, the sales of bioink in the global market were approximately USD 70 million [10]. Most of the published works are also limited in the use of hydrogels as bioink, mostly focusing on alginate, gelatin, and hyaluronic acid. The use of hydrogel biological ink can be molded during or immediately after printing. It is easy to create a 3D structure with a preferred shape and form, and its appropriate mechanical properties can control cell behavior. However, hydrogels also have some limitations as a bioinks, such as the poor mechanical strength of gelatin and the need to be exposed to ultraviolet light when methacrylic gelatin is covalently crosslinked, which may be detrimental to cell survival [133]. Currently, there are relatively few types of hydrogels that can be selected as bioinks, and different 3D printing methods also have different requirements for the properties of hydrogels. In 3D bioprinting, soft hydrogels are good for cell survival and differentiation, but it is difficult to maintain larger structures. It is still challenging to develop new biological inks and their additives that have biocompatibility and lead to shear thinning behavior, appropriate mechanical strength, printability, and fidelity. Therefore, it is also important for the design and synthesis of hydrogels as bioinks. With the development of artificial intelligence technology, the increasing demand, and continuous advancement of technology, the further development of hydrogel functions and the application prospects of 3D printing hydrogels in the field of tissue engineering will continue to increase, which is also an opportunity.

## Figures and Tables

**Table 1:** Types of 3D bioprinting technologies and their advantages and disadvantages.

3D Bioprinting Technologies	Advantages	Disadvantages	References
Inkjet bioprinting	Noncontact, easy, low cost, high cell vitality, and high speed	Few materials, low driving pressure, low printing accuracy, and small-size structures	[37,40]
Extrusion bioprinting	Wide range of materials, low cost, simple process and easy to use, good printability and fidelity, and large-size structures with preferred shapes and forms	Nozzle blockages, longer print times, low cell viability and missing materials, and layer-by-layer deposition limitations	[44,45,46]
Laser-assisted bioprinting	No nozzles, high resolution, automation, high cell vitality, and high repeatability and efficiency	The workstation is complex and requires a laser source	[47,48]
Stereolithography bioprinting	High print resolution, large-scale tissue, rapid printing, and high cell viability	Limited ability to capture the spatial heterogeneity	[50,51]
Suspension bioprinting	Stabilizes the gel form, maintains cell viability, broadens the application range of printing materials, and can encapsulate very-low-viscosity bioinks	The printing temperature is determined by the temperature of the suspension medium	[55,56]
Digital 3D bioprinting technologies	Higher spatial resolution, simple, faster print times, better cell viability, and can perform noninvasive 3D biological printing of tissue structures in vivo	More complex workstations, high-precision instruments, composite functional hydrogel biological ink materials	[56,60]

**Table 2:** Biological properties of 3D bioprinting hydrogels loaded with different cells or factors.

Cells/Factors	Hydrogel Composition	Target Tissue	Cellular Response	Bioprinting Method	Ref.
CCD-986Sk cells	SA (2.5%)-XG(6%) @cCNCs (55%)	Skin	>80% cell viability, up to day 14	Extrusion bioprinting	[80]
Human foreskin fibroblast cells	Methylcellulose/Alginate(2%)	Skin	>90% cell viability, up to day 7, high metabolic activity	Extrusion bioprinting	[81]
FBs, HUVECs, DPCs, EPCs	Gelatin (20%)-alginate (3%)	Skin	>80% cell viability, up to day 7, HF regeneration	Extrusion bioprinting	[18]
HUVECs	GelMA (10%)	Vessels	>90% cell viability, high proliferation rate, endothelial cell functionalization	Digital 3D bioprinting technologies	[82]
HUVECs, hBMSC	Alginate (1%), fibrin (30 mg/mL) and GelMA (5%)	Vascularisation and bone formation	VEGF, FGF-2, ANG-1 and EGF increased, cell differentiation	Extrusion bioprinting	[83]
Platelet lysate	PL (160 mgmL^−1)^-CNC (2.88%)	Tissues and organ surrogates	High cell viability, showing exceptionally fast spreading, growth, and synthesis of new ECM	Extrusion bioprinting	[84]
IL-4, MSCs	GelMA (20%)-dex (10%)	Skin	Excellent cytocompatibility, IL-4 and MSCs can synergistically induce macrophage polarization towards an anti-inflammatory M2 phenotype	Extrusion bioprinting, digital 3D bioprinting technologies	[85]
hMSCs	PEG (10%)-GelMA (1.5%)	Bones	>80% cell viability, the printed stem cells exhibit strong osteogenic and chondrogenic differentiation abilities	Inkjet bioprinting	[86]
hiPSC-MSCs, hSMCs	PEGDA (3%)-GelMA (7%)	Vessels	>80% cell viability, cell viability and physiological functions can be maintained at a high level	Stereolithography bioprinting	[50]
hiPSCs	HA(15%)	Tissuea and organa	Higher cell viability	Laser-assisted bioprinting	[87]
HUVECs	HA (3%)	Cell scaffolds	>90% cell viability, up to day 7	Suspension bioprinting	[54]

## Data Availability

Not applicable.
